# Bile acids at neutral and acidic pH induce apoptosis and gene cleavages in nasopharyngeal epithelial cells: implications in chromosome rearrangement

**DOI:** 10.1186/s12885-018-4327-4

**Published:** 2018-04-12

**Authors:** Sang-Nee Tan, Sai-Peng Sim

**Affiliations:** Department of Paraclinical Sciences, Faculty of Medicine and Health Sciences, Universiti Malaysia Sarawak, Sarawak, Malaysia

**Keywords:** CRS, NPC, Nasopharyngeal reflux, BA, Apoptosis, AF9, CAD

## Abstract

**Background:**

Chronic rhinosinusitis (CRS) increases the risk of developing nasopharyngeal carcinoma (NPC) while nasopharyngeal reflux is known to be one of the major aetiological factors of CRS. Bile acid (BA), the component of gastric duodenal contents, has been recognised as a carcinogen. BA-induced apoptosis was suggested to be involved in human malignancies. Cells have the potential and tendency to survive apoptosis. However, cells that evade apoptosis upon erroneous DNA repair may carry chromosome rearrangements. Apoptotic nuclease, caspase-activated deoxyribonuclease (CAD) has been implicated in mediating translocation in leukaemia. We hypothesised that BA-induced apoptosis may cause chromosome breaks mediated by CAD leading to chromosome rearrangement in NPC. This study targeted the *AF9* gene located at 9p22 because 9p22 is one of the most common deletion sites in NPC.

**Methods:**

We tested the ability of BA at neutral and acidic pH in inducing phosphatidylserine (PS) externalisation, reactive oxygen species (ROS) production, mitochondrial membrane potential (MMP) disruption, and caspase 3/7 activity in normal nasopharyngeal epithelial (NP69) and NPC (TWO4) cells. Inverse-PCR (IPCR) was employed to detect *AF9* gene cleavages. To investigate the role of CAD in mediating these cleavages, caspase inhibition was performed. IPCR bands representing *AF9* cleaved fragments were sequenced.

**Results:**

BA-treated cells showed higher levels of PS externalisation, ROS production, MMP loss and caspase 3/7 activity than untreated control cells. The effect of BA in the induction of these intracellular events was enhanced by acid. BA at neutral and acidic pH also induced significant cleavage of the *AF9* gene. These BA-induced gene cleavages were inhibited by Z-DEVD-FMK, a caspase-3 inhibitor. Intriguingly, a few chromosome breaks were identified within the *AF9* region that was previously reported to participate in reciprocal translocation between the mixed lineage leukaemia (*MLL)* and *AF9* genes in an acute lymphoblastic leukaemia (ALL) patient.

**Conclusions:**

These findings suggest a role for BA-induced apoptosis in mediating chromosome rearrangements in NPC. In addition, CAD may be a key player in chromosome cleavages mediated by BA-induced apoptosis. Persistent exposure of sinonasal tract to gastric duodenal refluxate may increase genomic instability in surviving cells.

**Electronic supplementary material:**

The online version of this article (10.1186/s12885-018-4327-4) contains supplementary material, which is available to authorized users.

## Background

Nasopharyngeal carcinoma (NPC) is a malignant tumour derived from the nasopharyngeal epithelium. The development of NPC is a multi-step process. NPC pathogenesis is related to Epstein-Barr virus (EBV) infection [1], consumption of preserved foods [2], exposures to wood dust and industrial heat [3], epigenetic changes [4] and genetic predisposition [5]. Moreover, chronic inflammation of sinonasal tract and/or ear (rhinitis, sinusitis, postnasal drip, otitis media) is increasingly being recognised as one of the risk factors for NPC [6–12]. More recently, a retrospective cohort study involving 231,490 Taiwan Chinese individuals revealed that individuals with chronic rhinosinusitis (CRS) have a 3.55-fold higher risk of developing NPC as compared with those without CRS [12]. Similar findings had been found in a case-control study which comprised 2242 NPC patients and 6726 controls; the odd ratio of prior CRS for subjects with NPC is 3.83 (95% confidence interval [CI], 3.23–4.53) [11]. The investigators attributed these findings to the chronic inflammation which may predispose the nasopharyngeal mucosa to transformation by carcinogens [7–9, 11, 12]. However, the causative factors and the underlying mechanisms which contribute to the chronic inflammation of nose or ear leading to NPC remain enigmatic.

As has been addressed by several reviews, chronic inflammation of sinonasal tract or ear is one of the extraoesophageal manifestations of gastro-oesophageal reflux disease (GORD) [13–17]. The flowing back of gastric duodenal contents into the oesophagus results in GORD. GORD is usually presented with symptoms such as heartburn and acid regurgitation. Apart from these classical symptoms, the gastric duodenal refluxate may flow beyond the oesophagus and affect the airways (tracheobronchopulmonary tree, larynx, pharynx, sinonasal tract and middle ear) [15]. These atypical manifestations of GORD are not only classified as extraoesophageal reflux (EOR) but also as laryngopharyngeal reflux, supra-oesophageal reflux, gastro-oesophago-pharyngeal reflux or gastro-oesophago-laryngeal reflux [15, 18]. It is sometimes called ‘silent reflux’ as more than half of the patients with EOR may not show ‘typical GORD’ symptoms (heartburn and acid regurgitation) [16].

GORD is an important factor in the pathogenesis of various inflammatory diseases. These diseases include gastritis [19, 20], oesophagitis [21–23], laryngitis [24–26], pharyngitis [27, 28], post-nasal drip [29], otitis media [30–33] and asthma [34–36]. Furthermore, CRS, which is the inflammation of the nose and paranasal sinuses, has also been included in this growing list [28, 37–39]. A high prevalence of GORD has been reported in children [40, 41] and adults [37, 42] with CRS. One possible underlying mechanism by which acid pharyngeal reflux may affect the sinonasal cavity is that, gastric duodenal contents reflux into the nasopharynx and the posterior nasal passages; direct contact of gastric duodenal contents on nasal mucosa causes inflammation and oedema. Subsequently, these phenomena lead to sinus ostial obstruction and sinusitis [43]. Reflux to the nasopharynx has been documented in both paediatric [40, 41, 44, 45] and adult groups [29, 37, 38, 46]. In addition, GORD treatment has improved or resolved the sinus symptoms in most patients with medically or surgically refractory CRS [46, 47].

Apart from being a risk factor for inflammatory disorders, GOR has also been implicated in several malignancies. Indeed, chronic inflammation has been well recognised as a strong factor in carcinogenesis [48]. GORD-related cancers include stomach cancer [49, 50], oesophageal adenocarcinoma [51, 52], laryngeal cancer [53], pharyngeal cancer [54] and lung cancer [55]. Previous studies have also shown that GOR is strongly linked to Barrett’s oesophagus [56, 57], a preneoplastic disorder of the oesophagus [58].

Acid refluxate consists of pancreatic fluids, hydrochloric acid, pepsin and bile acids (BA) [59]. BA has been recognised as a carcinogen in malignancies of the gastrointestinal tract. These include malignancies of the oesophagus, stomach, small intestine, liver, biliary tract, pancreas, colon and rectum (reviewed in [60]). BA has also been known to be a potent apoptotic inducer in human colon adenocarcinoma cells [61] and human oesophageal adenocarcinoma cells [62]. Furthermore, BA-induced apoptosis has been well implicated in the pathogenesis of Barrett’s oesophagus, oesophagus adenocarcinoma and colon cancer [63–65].

Apoptosis is a type of genetically controlled cell suicide programme [66]. In non-apoptotic cells, caspase-activated deoxyribonuclease (CAD) exists as a heterodimer with the inhibitor of CAD (ICAD). ICAD possess two caspase-3 cleavage sites. When caspase-3 is activated by apoptotic stimuli, it cleaves ICAD at the two caspase-3 cleavage sites and thus releases CAD [67, 68]. This allows CAD to oligomerise and form a large functional complex which cleaves DNA [69].

Although apoptosis is a programmed cell death process, cells may recover through DNA repair [70]. However, erroneous DNA repair may cause cells that survive apoptosis to harbour certain types of chromosome rearrangements, such as chromosome translocation and deletion [71]. The initial event of chromosome rearrangement is chromosome breakage. The apoptotic nuclease CAD has been found to be involved in these chromosome breakage events in both leukaemia [70, 72–74] and NPC [75–77]. Based on the literature, GORD is a contributing factor to CRS while CRS is a precursor of NPC. At the same time, BA from GORD can induce oxidative stress as well as apoptosis. Oxidative stress can also induce apoptosis resulting in chromosome breaks in cultured nasopharyngeal epithelial cells [77]. Since CAD has been implicated in mediating the chromosome breakage events in both leukaemia and NPC chromosome rearrangements, we thus hypothesise that BA-induced apoptosis may cause chromosome breaks by CAD leading to chromosome rearrangement in NPC, and this process may involve induction of oxidative stress. This study focuses on the *AF9* gene which is located at 9p22 because 9p22 is one of the deletion hotspots in NPC [78].

In this study, we report that BA induced PS externalisation, an early event of apoptosis, in normal nasopharyngeal epithelial and NPC cells. We demonstrated that BA-induced apoptosis triggered mitochondrial membrane potential (MMP) disruption, increased oxidative stress and activated caspase. Our findings also showed that these intracellular events were enhanced by acid. We further demonstrated that BA-induced apoptosis resulted in chromosome breaks within the *AF9* gene. These chromosome breaks were inhibited by caspase inhibitor (CI), suggesting that CAD may be the major player in mediating these chromosome breaks. Interestingly, a few breakpoints were the same as those reported in the mixed lineage leukaemia (*MLL*)-*AF9* fusion gene in an acute lymphoblastic leukaemia (ALL) patient. Lastly, we propose a potential schema for BA-induced apoptosis in mediating the chromosome breakages leading to chromosome rearrangements in NPC.

## Methods

### Cell line and chemicals

NP69 normal nasopharyngeal epithelial cell line was a kind gift from Prof. Tsao Sai Wah (The University of Hong Kong, Hong Kong, China) and Prof. Lo Kwok Wai (The Chinese University of Hong Kong, Hong Kong, China). TWO4 NPC cell line was a generous gift from Prof. Sam Choon Kook (formerly from University of Malaya, Malaysia). NP69 is an immortalised nasopharyngeal epithelial cell line which was established by transfection with SV40 large T oncogene. It retains some characteristics of normal nasopharyngeal epithelial cells and is non-tumourigenic. This cell line may provide potential nasopharyngeal epithelial cell model for studying mechanisms involved in the tumourigenesis of NPC [79]. TWO4 was derived from an undifferentiated NPC (WHO Type II B) of a 36-year-old Chinese female patient living in Taiwan [80].

Keratinocyte-SFM medium, RPMI 1640 medium, fetal bovine serum, L-glutamine, penicillin/streptomycin and StemPro ACCUTASE Cell Dissociation Reagent were procured from GIBCO, Invitrogen, USA. Taurocholic acid sodium salt hydrate, sodium glycochenodeoxycholate, glycocholic acid sodium, sodium deoxycholate, sodium glycodeoxycholate, dibasic sodium phosphate and citric acid were bought from Sigma, USA. Caspase-3 inhibitor II (Z-DEVD-FMK) was obtained from Calbiochem, USA. Camptothecin (CPT) was purchased from Santa Cruz Biotechnology, California, USA. 2′,7′-Dichlorofluorescein diacetate (DCFH-DA) was bought from Sigma-Aldrich, Israel. Annexin V-Fluorescein isothiocyanate (FITC) Apoptosis Detection Kit I and Flow Cytometry Mitochondrial Membrane Potential Detection Kit were purchased from Becton Dickinson Biosciences, USA. Caspase-Glo 3/7 Assay Kit was bought from Calbiochem, USA. QIAquick Nucleotide Removal Kit and QIAquick Gel Extraction Kit were procured from QIAGEN, Germany. Ammonium acetate was obtained from Merck, Germany. Phenol and Sodium dodecyl sulfate (SDS) were procured from Amresco, USA. Chloroform was obtained from R&M Chemicals, UK. Isoamyl alcohol was purchased from Fluka, Switzerland. DNA Polymerase I, Large (Klenow) Fragment, T4 DNA Ligase and all the restriction enzymes were obtained from New England Biolabs (NEB), USA. Phusion High-Fidelity DNA Polymerase was purchased from Finnzymes, Finland. PCR primers were purchased from First Base Laboratories. dNTP mix was bought from Promega, USA.

### Cell cultures

NP69 cells were grown in Keratinocyte-SFM medium supplemented with 100 U/ml penicillin, 100 μg/ml streptomycin, 2% (*v*/v) heat-inactivated fetal bovine serum, 4–5 ng/ml recombinant Epidermal Growth Factor (rEGF) and 40–50 μg/ml Bovine Pituitary Extract (BPE). TWO4 cells were cultured in RPMI 1640 medium supplemented with 100 U/ml penicillin, 100 μg/ml streptomycin, 10% (*v*/v) heat-inactivated fetal bovine serum and 2 mM L-glutamine. Cells were incubated at 37 °C with 5% CO_2_.

### Preparations of BA cocktail and acidified media

The BA cocktail was prepared according to the studies of Dvorak and colleagues [62]. It consists of an equimolar mixture of sodium salts of glycocholic acid, taurocholic acid, glycodeoxycholic acid, glycochenodeoxycholic acid and deoxycholic acid. The BA cocktail used in this study reflects the mixture of BA to which the distal oesophagus is exposed to during GOR [81–83]. Total BA concentrations commonly observed in the refluxate of patients with Barrett’s oesophagus fall within the range from 0.03 to 0.82 mM [82]. BA concentrations beyond this range (as high as 7.6 mM) have also been reported in the refluxate of some patients with Barrett’s oesophagus [84]. Therefore, concentrations in the physiological range were used in our studies (from 0.5 to 1.0 mM).

Furthermore, nasopharyngeal pH variations (pH below 4, 5 or 6) were observed in patients with GORD-related nasopharyngitis, CRS and otitis media [38, 41, 44, 85]. In a 24-h pH monitoring study, acidic nasopharyngeal pH was observed in GOR patients with chronic respiratory disease (otitis, sinusitis, laryngitis, epiglottitis, recurrent stridor, asthma and recurrent pneumonia). Nasopharyngeal pH of 5.8 had been considered as the best cut-off point to indicate the presence of abnormal pH-metry in GOR patients with chronic respiratory diseases [85]. Thus, in our study, we tested the apoptosis-inducing effect of BA in NP69 and TWO4 cells at neutral pH (pH 7.4) and acidic pH (pH 5.8). The media used in BA treatment at acidic pH were acidified to pH 5.8 with citrate phosphate buffer.

### Flow cytometric analysis of phosphatidylserine (PS) externalisation

NP69 cells (1 × 10^5^) and TWO4 cells (1.5 × 10^5^) were plated in 150 mm culture dishes and allowed to grow for 2 days. NP69 cells were either left untreated or treated with 0.5 mM of BA cocktail at pH 7.4 and pH 5.8 for 1 h. NP69 cells treated with 1.0 mM of CPT for 1 h were used to serve as a positive control. TWO4 cells were either left untreated or treated with 0.5 mM of BA at pH 7.4 and pH 5.8 for 3 h. TWO4 cells treated with 1.0 mM of CPT for 3 h were included as a positive control. CPT is a well-known apoptotic inducer. It has been shown that 2–10 μM of CPT with an exposure time of 24 h can induce apoptosis in NPC cells [86, 87]. After exposure, StemPro ACCUTASE Cell Dissociation Reagent was used to collect the cells. Using Annexin V-FITC Apoptosis Detection Kit I, the harvested cells were subjected to analysis of PS externalisation by a flow cytometer (FACSCalibur, Becton–Dickinson, USA) as previously described [77]. This analysis was performed in duplicate.

### Measurement of reactive oxygen species (ROS)

The intracellular ROS was determined by employing 2′,7′-dichlorofluorescein diacetate (DCFH-DA), a nonpolar, cell-permeable fluorescent probe described by Bass et al. (1983). DCFH-DA is nonfluorescent until hydrolysed by intracellular esterase and oxidised by ROS. Upon cleavage of the acetate groups by intracellular esterase, DCFH-DA is hydrolysed to polar dichlorofluorescein (DCFH) which remains trapped in viable cells. The nonfluorescent DCFH is rapidly oxidised to highly fluorescent dichlorofluorescein (DCF) in the presence of ROS [88]. NP69 (2 × 10^3^ cells per well) and TWO4 cells (3 × 10^3^ cells per well) were seeded in a 96-well black plate and allowed to adhere overnight. NP69 cells were left untreated or treated with 0.5 and 1.0 mM of BA cocktail at pH 7.4 and pH 5.8 for 1 h. NP69 cells treated with 20 mM of H_2_O_2_ for 1 h were used as a positive control. TWO4 cells were left untreated or treated with 0.5 and 1.0 mM of BA cocktail at pH 7.4 and pH 5.8 for 3 h. TWO4 cells treated with 5 mM of H_2_O_2_ for 3 h were included as a positive control. After exposure, media was removed and the cells were incubated with media containing 10 μM DCFH-DA for 30 min at 37 °C. The cells were then washed once with culture media. Fluorescence intensity was measured using a microplate reader (Tecan Infinite 200 Pro, Austria), with excitation at 485 nm and emission at 538 nm. This assay was carried out in triplicate. The cells were then subjected to fluorescence microscopic analysis under an inverted fluorescence microscope (IX 71, Olympus, Japan). Morphology of cells was photographed using microscope digital camera (DP72, Olympus, Japan).

### Flow cytometric analysis of mitochondrial membrane potential (MMP) disruption

NP69 cells (1 × 10^5^) and TWO4 cells (1.5 × 10^5^) were plated in 150 mm culture dishes and allowed to grow for 2 days. NP69 cells were either left untreated or treated with 0.5 mM of BA cocktail at pH 7.4 and pH 5.8 for 1 h. NP69 cells treated with 1.0 mM of CPT for 1 h were used to serve as a positive control. TWO4 cells were either left untreated or treated with 0.5 mM of BA at pH 7.4 and pH 5.8 for 3 h. TWO4 cells treated with 1.0 mM of CPT for 3 h were included as a positive control. Using Flow Cytometry Mitochondrial Membrane Potential Detection Kit, the collected cells were subjected to analysis of MMP disruption by a flow cytometer (FACSCalibur, Becton–Dickinson, USA) as previously described [77]. This assay was carried out in duplicate.

### Measurement of Caspase-3/7 activity

NP69 (2 × 10^3^ cells per well) and TWO4 cells (3 × 10^3^ cells per well) were seeded in a white-walled 96-well plate and allowed to adhere overnight. The NP69 and TWO4 cells were either left untreated or pretreated with 50 μM of Z-DEVD-FMK (Caspase-3 Inhibitor II) for 1 h. Subsequently, the NP69 cells were either left untreated or cotreated for 1 h with 0.5 mM of BA cocktail, at pH 7.4 and pH 5.8, while the TWO4 cells were left untreated or cotreated for 3 h with 0.5 mM of BA cocktail, at pH 7.4 and pH 5.8. After exposure, the media was removed and the cells were washed once with 1 x PBS buffer. Using Caspase-Glo 3/7 Assay Kit (Promega), caspase-3/7 activity was determined by a microplate reader (Tecan Infinite 200 Pro, Austria) as previously described [77]. This assay was carried out in duplicate.

### Identification of BA-induced chromosome breaks by nested inverse polymerase chain reaction (IPCR)

#### Apoptosis induction assay

NP69 cells (1.5 × 10^4^) and TWO4 cells (2.5 × 10^4^) were plated in 60 mm culture dishes and allowed to grow for 2 days. NP69 cells were left untreated or treated with 0.5 mM of BA cocktail at pH 7.4 and pH 5.8 for 1 h. TWO4 cells were left untreated or treated with 0.5 mM of BA cocktail at pH 7.4 and pH 5.8 for 3 h.

#### Genomic DNA extraction

Genomic DNA extraction was carried out using phenol/chloroform/isoamyl alcohol extraction method as previously described [77].

#### Manipulation of genomic DNA in preparation for nested IPCR

The extracted genomic DNA was manipulated for nested IPCR as previously described [77]. These manipulation steps were depicted in Additional file 1.

#### Nested IPCR

Using an ultraviolet-visible microvolume spectrophotometer (ND-1000, NanoDrop, USA), optical density (O.D.) of the purified DNA sample was determined. Nested IPCR was performed as previously described [77]. All the IPCR amplifications were carried out using a Veriti 96 Well Thermal Cycler (Applied Biosystems, USA).

### Caspase inhibition assay

NP69 cells (1.5 × 10^4^) were plated in 60 mm culture dishes and allowed to grow for 2 days. NP69 cells were either left untreated or pretreated with 50 μM of Z-DEVD-FMK for 1 h. Following that, the NP69 cells were either left untreated or cotreated with 0.5 mM of BA at pH 7.4 and pH 5.8 for 1 h. Subsequently, genomic DNA extraction and IPCR detection of the chromosome breaks were performed as described above.

### Agarose gel electrophoresis and DNA sequencing of the cleavage bands

In order to visualise cleaved chromosomes, the IPCR products were analysed on 1% agarose gel and stained with ethidium bromide. To map the breakpoints, IPCR bands representing the *AF9* cleaved fragments were excised, cleaned up with QIAGEN QIAquick Gel Extraction Kit and sequenced. The sequencing results were then annotated by blasting the human genome database (Nucleotide BLAST, http://blast.ncbi.nlm.nih.gov/Blast.cgi). By aligning the sequencing data with the *AF9* gene sequence retrieved from Ensembl database [EMBL:ENSG00000171843] using Seqman DNASTAR software (Lasergene, USA), the breakpoints of the *AF9* cleaved fragments were determined. A genomic map illustrating the breakpoints was constructed.

### Quantification of gene cleavage frequency

IPCR amplification was performed in two to five sets per experiment. Each set of IPCR consisted of three to six replicates per cell sample. The gene cleavage frequencies represent the average number of the *AF9* cleaved fragments detected in at least three independent experiments.

### Statistical analysis

Data were presented as means with standard deviation (SD). Student’s *t*-test was used to evaluate the significance of differences between the untreated control and the treated groups in flow cytometric analyses of PS externalisation and MMP loss, determination of ROS level, measurement of caspase activity and IPCR assays. All statistical tests were two-sided. Differences were considered statistically significant at *p*-value < 0.05.

## Results

### Bile acid induces apoptosis

#### Bile acid induces phosphatidylserine externalisation

**Fig. 1:**
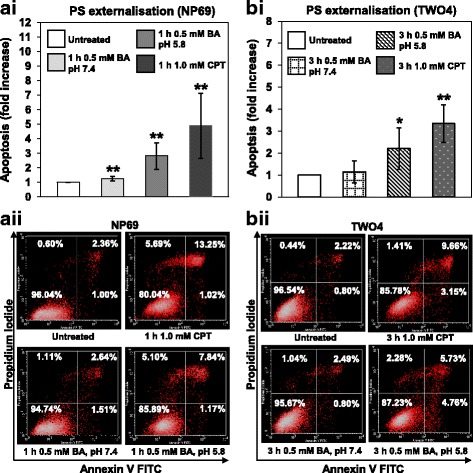
Bile acid induces phosphatidylserine externalisation in NP69 and TWO4 cells. NP69 cells were either left untreated or treated for 1 h with 0.5 mM of BA at pH 7.4 and pH 5.8. TWO4 cells were either left untreated or treated for 3 h with 0.5 mM of BA at pH 7.4 and pH 5.8. CPT was used as a positive control. After exposure, flow cytometric analysis of PS externalisation was performed in (**a i**) NP69 and (**b i**) TWO4 cells. Means and SD of three independent experiments performed in duplicate are shown. Data are expressed as fold increase normalised to the untreated control. Student’s *t-*test was used for statistical analysis to compare treated groups with the untreated control, * *p* < 0.05, ** *p* < 0.01. Representative dot plot diagrams showing the apoptotic populations of BA-treated (**a ii**) NP69 and (**b ii**) TWO4 cells detected by Annexin V-FITC and PI staining are shown. The lower left quadrants show viable cells; the lower right quadrants represent early apoptotic cells; the upper right quadrants show late apoptotic and necrotic cells

The potential role of BA in inducing apoptosis in NP69 and TWO4 cells was examined by flow cytometric analysis of PS externalisation. NP69 cells were treated with 0.5 mM of BA at pH 7.4 and pH 5.8 for 1 h. The percentages of apoptotic cells are 1.2-fold (*p*-value = 0.009) and 2.8-fold (*p*-value = 0.005) higher than that of the untreated control, for pH 7.4 and pH 5.8, respectively (Fig. 1a i). Similarly, TWO4 cells were treated with 0.5 mM of BA at pH 5.8 for 3 h. The percentage of apoptotic cells is 2.2-fold (*p*-value = 0.026) higher than that of the untreated control. However, only a minimal percentage of apoptotic cells were detected in TWO4 cells treated with 0.5 mM of BA at pH 7.4 for 3 h (*p*-value = 0.541) (Fig. 1b i). Camptothecin (CPT) was used as a positive control. Figures 1a ii and b ii show representative dot plot diagrams indicating the apoptotic populations of BA-treated NP69 and TWO4 cells. Taken together, these data indicate that the combination of BA and acid has a higher apoptotic effect in nasopharyngeal epithelial cells as compared with BA alone.

#### Bile acid induces mitochondrial membrane potential (MMP) disruption

**Fig. 2:**
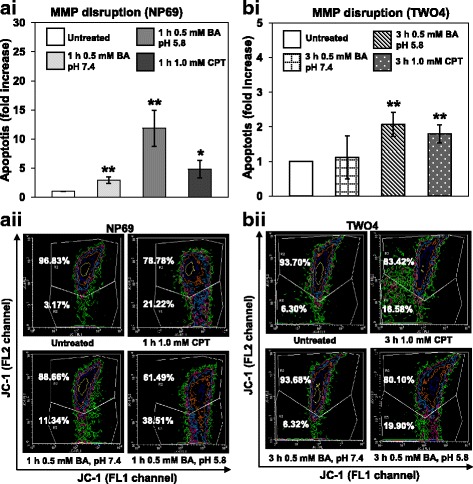
Bile acid induces mitochondrial membrane potential disruption in NP69 and TWO4 cells. NP69 cells were either left untreated or treated for 1 h with 0.5 mM of BA at pH 7.4 and pH 5.8. TWO4 cells were either left untreated or treated for 3 h with 0.5 mM of BA at pH 7.4 and pH 5.8. CPT was used as a positive control. After incubation, MMP disruption was assessed by flow cytometry in (**a i**) NP69 and (**b i**) TWO4 cells. Data presented are expressed as means and SD of two independent experiments performed in duplicate. The differences between untreated control and treated groups were compared by using Student’s *t*-test, * *p* < 0.05, ** *p* < 0.01. Representative contour plot diagrams showing the apoptotic populations of BA-treated NP69 (**a ii**) and TWO4 cells (**b ii**) determined by JC-1 staining are shown. The upper and lower quadrants represent the percentages of viable cells and apoptotic cells, respectively

Changes in MMP were assessed in BA-treated cells using flow cytometry. In NP69 treated for 1 h with 0.5 mM of BA at pH 7.4 and pH 5.8, the percentages of population with MMP disruption are 2.9-fold (*p*-value = 0.007) and 11.8-fold (*p*-value = 0.006) higher than that of the untreated control, respectively (Fig. 2a i). As shown in Fig. 2b i, the percentage of population with MMP loss in TWO4 cells treated with 0.5 mM of BA at pH 5.8 for 3 h is 2.1-fold (*p*-value = 0.009) higher than that in the untreated control. There was no significant change in MMP disruption when TWO4 cells were treated with 0.5 mM of BA at neutral pH (*p*-value = 0.737). Treatment of NP69 and TWO4 cells with CPT were used as positive controls. Figures 2a ii and b ii show representative contour plot diagrams indicating the populations with disruption of MMP in BA-treated NP69 and TWO4 cells. Collectively, these results show that the combination of BA and acid has a higher effect in triggering MMP loss in nasopharyngeal epithelial cells as compared with BA alone.

#### Bile acid-induced apoptosis involves caspase activity that is reduced by a caspase inhibitor

**Fig. 3:**
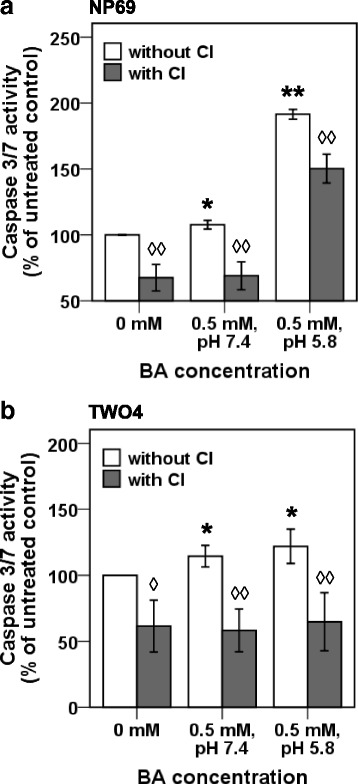
Bile acid-induced apoptosis involves caspase activity that is reduced by a caspase inhibitor. NP69 and TWO4 cells were left untreated or pretreated with 50 μM of Z-DEVD-FMK (Caspase-3 Inhibitor II) for 1 h. Subsequently, NP69 cells were treated with 0.5 mM of BA at pH 7.4 and pH 5.8 for 1 h, whereas TWO4 were treated with 0.5 mM of BA at pH 7.4 and pH 5.8 for 3 h. The activity of caspase-3/7 was measured by using Caspase-Glo 3/7 Assay in (**a**) NP69 and (**b**) TWO4 cells. Means and SD of two independent experiments performed in duplicate are shown. Values were normalised to the percentage of untreated control. The differences between the untreated control and treated groups were compared by using Student’s *t*-test. An asterisk indicates a significant increase in BA-treated cells as compared with the untreated control, * *p* < 0.05, ** *p* < 0.01. An open diamond indicates a significant decrease in the sample with CI pretreatment as compared with its corresponding sample without CI pretreatment, ◊ *p* < 0.05, ◊◊ *p* < 0.01

To examine if BA-induced apoptosis is caspase-dependent, the activity of effector caspases, caspase-3 and caspase-7, was determined in BA-treated NP69 and TWO4 cells. NP69 cells treated with 0.5 mM of BA at pH 7.4 and pH 5.8 for 1 h showed 8% (*p*-value = 0.018) and 92% (*p*-value < 0.001) higher caspase 3/7 activities than that in the untreated control, respectively (Fig. 3a). The caspase 3/7 activities of TWO4 cells are 15% (*p*-value = 0.038) and 22% (*p*-value = 0.043) higher than that of the untreated control after 3 h treatment with 0.5 mM of BA at pH 7.4 and pH 5.8, respectively (Fig. 3b). Pretreatment with Caspase-3 Inhibitor II, Z-DEVD-FMK, significantly reduced caspase 3/7 activity in BA-cotreated NP69 and TWO4 cells. These findings suggest that caspase-3 and caspase-7 are involved in BA-induced apoptosis at both neutral and acidic pH.

### Bile acid triggers production of intracellular reactive oxygen species (ROS)

**Fig. 4:**
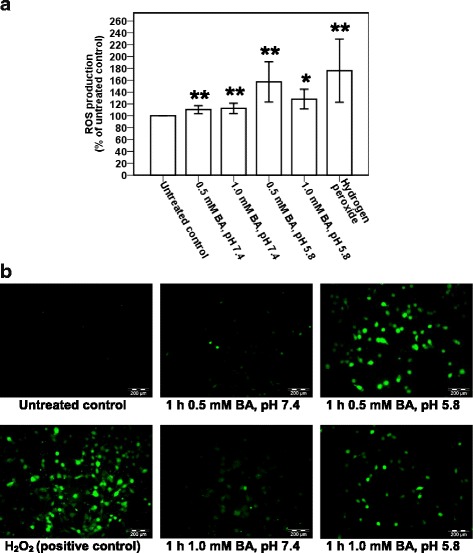
Bile acid triggers production of reactive oxygen species in NP69 cells. NP69 cells were treated with 0.5 and 1.0 mM of BA at pH 7.4 and pH 5.8 for 1 h. 20 mM of H_2_O_2_ was used as a positive control. After incubation with DCFH-DA, the cells were subjected to determination of ROS level and fluorescence microscopic analysis as described in “Methods” section. **a** Percentages of increased ROS level in NP69 cells after treatment with BA. Data are means and SD of five independent experiments performed in triplicate. Values are expressed in percentages with respect to values obtained from the untreated control. The differences between the untreated control and treated groups were compared by using Student’s *t*-test, * *p* < 0.01, ** *p* < 0.001. **b** Representative fluorescence microscopic images showing the BA-treated NP69 cells are shown. Magnification 100X, bar = 200 μm

**Fig. 5:**
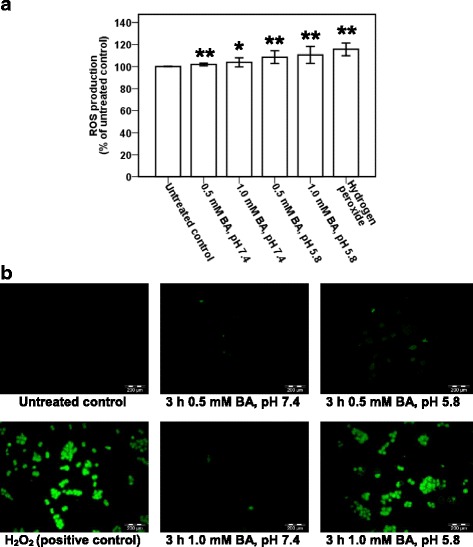
Bile acid triggers production of reactive oxygen species in TWO4 cells. TWO4 cells were treated with 0.5 and 1.0 mM of BA at pH 7.4 and pH 5.8 for 3 h. 5 mM of H_2_O_2_ was included as a positive control. After exposure, ROS determination and fluorescence microscopy analysis were performed as described in “Methods” section. **a** Percentages of increased ROS generation in BA-treated TWO4 cells. Data are means and SD of five independent experiments carried out in triplicate. Values are presented in percentages with respect to values obtained from the untreated control. The differences between the untreated control and treated groups were compared by using Student’s *t*-test, * *p* < 0.01, ** *p* < 0.001. **b** Representative fluorescence microscopic images showing the BA-treated TWO4 cells are shown. Magnification 100X, bar = 200 μm

In order to investigate if BA induces intracellular ROS generation, the BA-treated NP69 and TWO4 cells were subjected to ROS measurement and fluorescence microscopic analysis. NP69 cells were treated for 1 h with 0.5 mM of BA at pH 7.4, 1.0 mM of BA at pH 7.4, 0.5 mM of BA at pH 5.8 and 1.0 mM of BA at pH 5.8; the ROS generated are 10% (*p*-value < 0.001), 13% (*p*-value < 0.001), 57% (*p*-value < 0.001) and 28% (*p*-value = 0.002) higher than that of the untreated control, respectively (Fig. 4a). In TWO4 cells treated for 3 h with 0.5 mM of BA at pH 7.4, 1.0 mM of BA at pH 7.4, 0.5 mM of BA at pH 5.8 and 1.0 mM of BA at pH 5.8, the ROS generated are 2% (*p*-value < 0.001), 4% (*p*-value = 0.002), 9% (*p*-value < 0.001) and 11% (*p*-value < 0.001) higher than that of the untreated control, respectively (Fig. 5a). A well-known inducer of oxidative stress, hydrogen peroxide (H_2_O_2_), was used as a positive control. Representative fluorescence microscopic images showing the BA-treated NP69 and TWO4 cells were shown in Figs. 4b and 5b. These findings suggest that exposure of nasopharyngeal epithelial cells to BA resulted in intracellular ROS generation. Furthermore, the effect of BA on the induction of oxidative stress was enhanced by acid.

### BA-induced apoptosis results in chromosome breaks in the *AF9* gene

To test if BA-induced apoptosis leads to cleavage in the *AF9* gene, BA-treated NP69 and TWO4 cells were subjected to genomic DNA extraction and subsequently IPCR. Two breakpoint cluster regions (BCR) have been reported in the *AF9* gene. These two BCRs were denominated as BCR1 and BCR2 [89, 90]. In the present study, nested IPCR was used to detect chromosome breaks within the *AF9* BCR1 which is located at the telomeric end of intron 4. Based on the primers position, the intact IPCR product is 944 bp (~ 950 bp). Consequently, IPCR bands smaller than 950 bp will be detected if there is any chromosome breakage within the region of study.

**Fig. 6:**
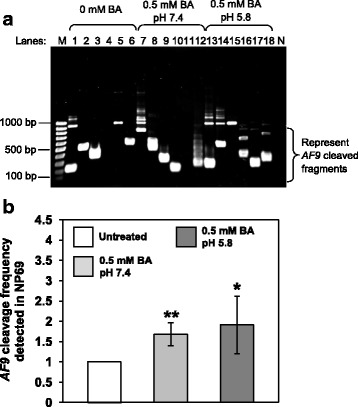
Bile acid induces *AF9* gene cleavages in NP69 cells. IPCR was employed to identify the *AF9* gene cleavages in NP69 cells after exposed to BA. **a** Representative gel picture showing the *AF9* gene cleavages identified by IPCR. NP69 cells were left untreated (Lanes 1–6) or treated for 1 h with 0.5 mM of BA at pH 7.4 (Lanes 7–12) and pH 5.8 (Lanes 13–18). Genomic DNA extraction and nested IPCR were performed as described in “Methods” section. The side bracket represents the IPCR bands derived from the *AF9* cleaved fragments. M: 100 bp DNA marker. N: negative control for IPCR. **b** Average number of the *AF9* gene cleavages identified in BA-treated NP69 cells. Data are expressed as means and SDs of four independent experiments. Each experiment consisted of two to five sets of IPCR carried out in three to six replicates per set for each cell sample. Values are expressed as fold change normalised to the value of the untreated control. The differences between the untreated control and treated groups were compared by using Student’s *t*-test, * *p* < 0.05, ** *p* < 0.01

**Fig. 7:**
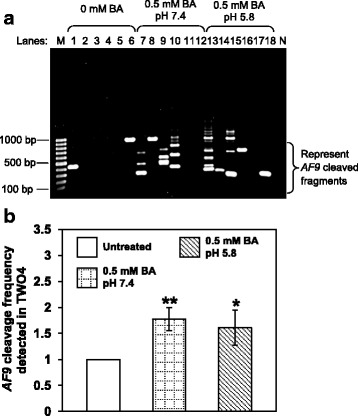
Bile acid induces *AF9* gene cleavages in TWO4 cells. Genomic DNA was extracted from BA-treated TWO4 cells for nested IPCR as described in “Methods” section. **a** Representative gel picture showing the *AF9* gene cleavages detected in BA-treated TWO4 cells. TWO4 cells were left untreated (Lanes 1–6) or treated for 3 h with 0.5 mM of BA at pH 7.4 (Lanes 7–12) and pH 5.8 (Lanes 13–18). The IPCR bands derived from the *AF9* cleaved fragments were indicated by the side bracket. M: 100 bp DNA ladder. N: Negative control for IPCR. **b** Average number of the *AF9* gene cleavages detected by IPCR. Data represents means and SDs of three independent experiments. Each experiment consisted of four sets of IPCR assays performed in three to six replicates per set for each cell sample. Values are expressed as fold change normalised to the value of the untreated control. The differences between the untreated control and treated groups were compared by using Student’s *t*-test, * *p* < 0.05, ** *p* < 0.01

The representative gel pictures indicating *AF9* gene cleavages detected in NP69 and TWO4 cells after treatment with BA at pH 7.4 and pH 5.8 are shown in Figs. 6a and 7a, respectively. As assessed by the flow cytometric detection of apoptosis, a minimal amount of dying cells was detected in the untreated sample. These dying cells might undergo spontaneous chromosome breakages contributing to the background. In NP69 cells treated with 0.5 mM of BA at pH 7.4 and pH 5.8 for 1 h, the *AF9* gene cleavage frequencies are 1.7-fold (*p*-value = 0.006) and 1.9-fold (*p*-value = 0.045) higher than that in the untreated cells, respectively (Fig. 6b). Similarly, in TWO4 cells treated with 0.5 mM of BA at pH 7.4 and pH 5.8 for 3 h, the gene cleavage frequencies are 1.8-fold (*p*-value = 0.004) and 1.6-fold (*p*-value = 0.036) higher than that in the untreated cells, respectively (Fig. 7b). Our data clearly indicate that BA-induced apoptosis in NP69 and TWO4 cells results in the *AF9* gene cleavages at both neutral and acidic pH.

### Caspase inhibitor reduces *AF9* gene cleavages in bile acid-cotreated NP69 cells

To investigate if CAD is responsible for mediating the chromosome cleavages during BA-induced apoptosis, caspase inhibition assay was conducted. In normal healthy cells, CAD exists naturally with its chaperone, ICAD, in the cytoplasm. When apoptosis is induced by its stimulus such as BA, caspase-3 will be triggered and cleaves the ICAD. CAD will be subsequently released from ICAD and enters the nucleus to cleave the chromosomal DNA [67, 68]. DEVD inhibitor was found to be the most effective caspase-3 inhibitor [91]. Thus, if CAD is responsible for mediating chromosome cleavages during BA-induced apoptosis, by inhibiting caspase-3 using DEVD inhibitor, chromosome breaks in BA-treated cells will be reduced, if not totally eliminated.

**Fig. 8:**
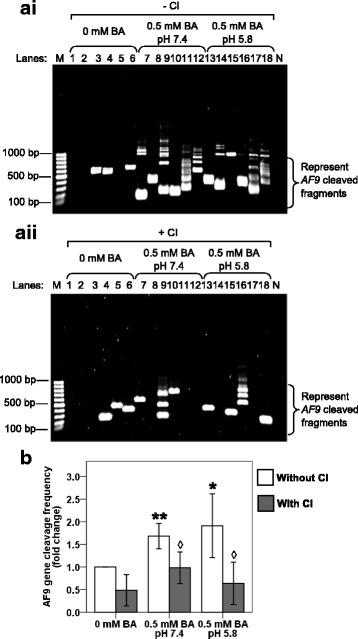
Caspase inhibitor reduces *AF9* gene cleavages in bile acid-cotreated NP69 cells. **a** Representative gel pictures showing the IPCR detection of the *AF9* gene cleavages in BA-treated NP69 cells: (**i**) without CI pretreatment (**ii**) with CI pretreatment. NP69 cells were left untreated or pretreated with 50 μM of Z-DEVD-FMK (Caspase-3 inhibitor II) for 1 h. The NP69 cells were subsequently left untreated (Lanes 1–6) or treated for 1 h with 0.5 mM of BA at pH 7.4 (Lanes 7–12) and pH 5.8 (Lanes 13–18). Genomic DNA was extracted and modified for nested IPCR as described in “Methods” section. The IPCR bands derived from the *AF9* cleaved fragments were indicated by the side brackets. **b** Average number of *AF9* gene cleavages detected in BA-treated NP69 cells. Data are presented as means and SDs of four independent experiments. Each experiment consisted of two to four sets of IPCR performed in three to six IPCR replicates per set for each cell sample. Values are expressed as fold change normalised to the value of the untreated control. The differences between the untreated control and treated groups were compared by using Student’s *t*-test. An asterisk indicates a significant increase in BA-treated cells as compared with the untreated control (* *p* < 0.05, ** *p* < 0.01). An open diamond represents a significant decrease in the sample with CI pretreatment as compared with its corresponding sample without CI pretreatment (*p* < 0.01)

NP69 cells were either pretreated or left untreated with Z-DEVD-FMK. Subsequently, the cells were either left untreated or cotreated with BA at neutral and acidic pH. After BA treatment, IPCR was performed as described above. The representative gel pictures showing the IPCR results of BA-treated NP69 cells in the absence (Fig. 8a i) and presence (Fig. 8a ii) of Z-DEVD-FMK were shown in Fig. 8. Pretreatment with Z-DEVD-FMK has significantly reduced the gene cleavage frequency for approximately 1.7-fold in NP69 cells treated with BA at neutral pH (*p*-value = 0.006) and 3.0-fold in NP69 cells treated with BA at acidic pH (*p*-value = 0.010) (Fig. 8b). These findings together suggest that CAD is an essential player in mediating the chromosome breaks triggered by BA.

### Sequencing results

**Fig. 9:**
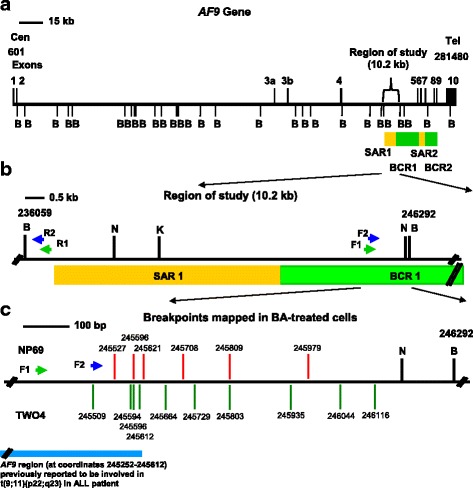
The positions of bile acid-induced chromosome breakages within the *AF9* gene. **a**The genomic map of *AF9* gene from nucleotide positions 601–281,480 is illustrated [EMBL:ENSG00000171843]. Green boxes represent the two previously identified patient breakpoint cluster regions which are denoted as BCR1 and BCR2 [89]. Yellow boxes indicate the biochemically defined MAR/SARs reported in the previous study. These two MAR/SARs were denominated as SAR1 and SAR2 [89]. B: *Bam*H I restriction sites. **b** Targeted region (10.2 kb) in the *AF9* gene. Green and blue arrows indicate the primers used in the first (R1, AF9 236,451 R and F1, AF9 245,385 F) and second (R2, AF9 236,211 R and F2, AF9 245,507 F) rounds of nested IPCR, respectively. *Bam*H I (B), *Kpn* I (K) and *Nde* I (N) restriction sites are shown. **c** Chromosome breakages detected in the present study by using IPCR. Red and green vertical lines indicate the presently identified breakpoints in BA-treated NP69 and TWO4 cells, respectively. All the chromosome breakages were mapped within BCR1 in close proximity with SAR1. Blue box represents the *AF9* region (at coordinates 245,252–245,612) that was previously reported to translocate with the *MLL* gene resulting in the *MLL-AF9* fusion gene in an ALL patient [GenBank:AM050804]

To ascertain that these fragments were derived from the cleaved *AF9* gene, the IPCR bands were cleaned up and sequenced. The sequencing results show that these fragments are all derived from the cleaved *AF9* gene. The positions of chromosome breaks detected within the *AF9* gene in BA-treated NP69 and TWO4 cells are shown in Tables 1 and 2, respectively. Intriguingly, four breakpoints (at coordinates 245,509, 245,527, 245,594 and 245,596) were mapped within the *AF9* region (at coordinates 245,252–245,612) which was previously reported to be involved in t(9;11)(p22;q23) in an ALL patient. This chromosome translocation resulted in the formation of the *MLL-AF9* fusion gene in the ALL patient [GenBank:AM050804]. It is noteworthy that in BA-treated TWO4 cells, we identified a breakpoint (at coordinate 245,612) which is identical with that in the ALL patient [GenBank:AM050804]. A breakpoint (at coordinate 245,596) was simultaneously identified in both NP69 and TWO4 cells treated with BA at neutral pH. Three breakpoints (at coordinates 245,594, 245,596 and 246,116) are similar with those identified in CEM cells (at coordinate 246,114) and cultured normal blood cells (at coordinate 245,593) exposed to etoposide (VP16) [74]. A few breakpoints (at coordinates 245,596, 245,664, 245,708, 245,803 and 246,116) are similar with those identified in H_2_O_2_-treated NP69 cells (at coordinates 245,591, 245,659, 245,703, 245,796, 246,113) and HK1 cells (at coordinate 245,590) reported in our previous study [77]. A genomic map illustrating the breakpoints within the *AF9* BCR in BA-treated NP69 and TWO4 cells is shown in Fig. 9.

**Table 1:** Breakpoints detected within the *AF9* gene in BA-treated NP69 cells

*BA-treated NP69 cells*	*Breakpoint*	*Remarks*
0.5 mM BA, pH 7.4	245527	This chromosome break falls within the *AF9* region (at coordinates 245,252–245,612) that was previously reported to translocate with the *MLL* gene leading to the formation of the *MLL*-*AF9* fusion gene in an ALL patient [GenBank:AM050804].
	245596	This chromosome break falls within the *AF9* region (at coordinates 245,252–245,612) that was previously reported being involved in the formation of the *MLL*-*AF9* fusion gene in an ALL patient. This breakpoint is three nucleotides different from that reported in cultured normal blood cells treated with VP16 (at coordinate 245,593) [74].
	245979	
0.5 mM BA, pH 5.8	245621	This breakpoint is nine nucleotides different from that identified in an ALL patient (at coordinate 245,612) [GenBank:AM050804].
	245708	This breakpoint is five nucleotides different from that reported in H_2_O_2_-treated NP69 cells (at coordinate 245,703) [77].
	245809	

The breakpoints identified within the *AF9* gene were mapped according to the *AF9* sequence retrieved from Ensembl database [EMBL:ENSG00000171843]

**Table 2:** Breakpoints detected within the *AF9* gene in BA-treated TWO4 cells

*BA-treated TWO4 cells*	*Breakpoint*	*Remarks*
0.5 mM BA, pH 7.4	245596	This breakpoint is identical with a breakpoint identified in NP69 treated with BA at pH 7.4. This chromosome break falls within the *AF9* region (at coordinates 245,252–245,612) previously reported being involved in the formation of the *MLL*-*AF9* fusion gene in an ALL patient [GenBank:AM050804]. This breakpoint is three nucleotides different from that reported in cultured normal blood cells treated with VP16 (at coordinate 245,593) [74], five nucleotides different from a breakpoint detected in H_2_O_2_-treated NP69 cells (at coordinate 245,591) and six nucleotides different from that identified in H_2_O_2_-treated HK1 cells (at coordinate 245,590) [77].
	245664	This breakpoint is five nucleotides different from a breakpoint (at coordinate 245,659) identified in NP69 cells treated with H_2_O_2_ [77].
	245803	This breakpoint is seven nucleotides different from a breakpoint (at coordinate 245,796) reported in NP69 cells treated with H_2_O_2_ [77].
	245935	
0.5 mM BA, pH 5.8	245509	This chromosome break falls within the *AF9* region (at coordinates 245,252–245,612) that was previously reported to translocate with the *MLL* gene leading to the formation of the *MLL*-*AF9* fusion gene in an ALL patient [GenBank:AM050804].
	245594	This chromosome break falls within the *AF9* region (at coordinates 245,252–245,612) that was previously reported to translocate with the *MLL* gene leading to the formation of the *MLL*-*AF9* fusion gene in an ALL patient [GenBank:AM050804]. This breakpoint is one nucleotide different from that reported in cultured normal blood cells treated with VP16 (at coordinate 245,593) [74], three nucleotides different from a breakpoint detected in H_2_O_2_-treated NP69 cells (at coordinate 245,591), and four nucleotides different from that identified in H_2_O_2_-treated HK1 cells (245590) [77].
	245612	This breakpoint is identical with the breakpoint previously identified in an ALL patient [GenBank:AM050804].
	245729	
	246044	
	246116	This breakpoint is two nucleotides different from a breakpoint (at coordinate 246,114) reported in CEM cells exposed to VP16 [74], and three nucleotides different from a breakpoint (at coordinate 246,113) identified in NP69 cells treated with H_2_O_2_ [77].

The breakpoints identified within the *AF9* gene were mapped according to the *AF9* sequence retrieved from Ensembl database [EMBL:ENSG00000171843]

## Discussion

In the present study, we intended to investigate the role of BA-induced apoptosis in mediating chromosome rearrangement in NPC. The externalisation of PS appears to be an early event of apoptosis [92]. Using flow cytometric analysis of PS externalisation, we observed significant percentages of apoptosis after BA treatment. This indicates that BA is a potential apoptotic inducer in nasopharyngeal epithelial cells. The results of this study reaffirm the findings of previous studies where BA could induce apoptosis in a number of cell types. These include normal oesophageal mucosal epithelial cells [93, 94], oesophageal adenocarcinoma cells [62, 95], Barrett epithelial cancer cells [96], normal colon epithelial cells [63], colon adenocarcinoma cells [61], hepatocellular carcinoma cells [97] and rat hepatocyte [98].

It is important to note that, BA at acidic pH resulted in a higher percentage of apoptotic cells as compared with that at neutral pH. This is true for both NP69 and TWO4 cells. Our findings are consistent with previous work which demonstrated that the combination of acid and BA has a higher apoptosis-inducing effect as compared with either acid alone or BA alone [62]. Therefore, it is likely that there is a synergistic effect between BA and acidic pH in apoptosis induction. Indeed, there is abundant evidence that the combination of acid and BA has a higher injurious effect on the epithelial cells as compared with either acid alone or BA alone [82, 99–101].

Evidence has been given that BA triggers apoptosis via oxidative stress [61]. It is known that BA can induce the generation of intracellular ROS in human colon epithelial cells [61, 102], human oesophageal epithelial cells [62, 101] and rat hepatocyte [98]. In the present study, we tested the ability of BA in inducing ROS generation in NP69 and TWO4 cells. By using a dichlorofluorescein diacetate (DCFH-DA) fluorescence-based assay, we have demonstrated that BA at neutral and acidic pH induced the production of ROS in both cell lines. Our findings are consistent with previous work which demonstrated that BA at neutral and acidic pH (pH 6) induced the release of ROS within the cytoplasm of exposed oesophageal adenocarcinoma cells (OE33) and oesophageal squamous cell carcinoma cells (KYSE-3) [101]. However, it is noteworthy that in both NP69 and TWO4 cells, the effect of BA in triggering ROS generation was higher at acidic pH than at neutral pH. This supports an earlier study of Dvorak and colleagues which suggested that there is a synergism between acid and BA in oxidative stress induction. In their study, a significant increase in oxidative stress was found in oesophageal adenocarcinoma cells (Seg-1) exposed to pH 4 in combination with BA cocktail, but not in Seg-1 cells treated with acid alone or BA alone. Their study also showed that generation of 8-OH-dG, a marker of oxidative DNA damage, was significantly increased in biopsy specimens after incubation in medium acidified to pH 4 and BA cocktail, but not in specimens treated with acid only or BA only [62]. The authors have therefore proposed a few possible mechanisms of synergism between acid and BA in oxidative stress induction. One of the possible mechanisms is that acid and BA synergise to activate nicotinamide adenine dinucleotide phosphate (NADPH) oxidase, through endosomal acidification by acid [103] and membrane perturbation by BA [102]. It has also been speculated that BA-induced ROS might be enhanced by acidic pH through iron-mediated Fenton reactions [104].

It is known that in BA-induced apoptosis, ROS is mainly generated through the activation of NADPH oxidases [61, 102]. However, it has also been observed that inhibition of NADPH oxidases activities did not completely protect BCS-TC2 cells from the cytotoxic effects of BA, indicating potential additional minor sources of cytotoxicity. Due to the hydrophobic nature of BA, BA may directly diffuse into the cytosol resulting in mitochondrial perturbations. This can lead to subsequent alteration in oxidative phosphorylation which causes excessive ROS formation [61]. This ROS generation is strongly associated with the onset of mitochondrial permeability transition (MPT) which is an important feature of BA-induced apoptosis [98]. One of the hallmarks of the induction of MPT is the loss of MMP [105].

In the current study, we have demonstrated that BA stimulated MMP disruption in nasopharyngeal epithelial cells. The capability of BA in stimulating MMP disruption, which represents MPT, has also been demonstrated in rat hepatocyte [98], human colon carcinoma tumourigenic cells (HCT-116) [102] and non-tumourigenic cells (BCS-TC2) [61]. In consistency with the ROS detection assay, the BA-triggered MMP loss was substantially enhanced by acidic pH. Since the collapse of MMP is an event greatly dependent on the signalling molecules, ROS, these findings therefore strengthen the suggestion that acid and BA synergise to stimulate oxidative stress (as discussed above).

Previous studies have shown that activation of MPT by oxidative stress is a crucial event for the downstream caspase activation and apoptosis. This BA-induced MPT can provoke the release of pro-apoptotic proteins (such as cytochrome *c*) to the cytosol leading to the activation of initiator caspase-9. Subsequently, effector caspase-3 is activated, followed by the activation of DNA degrading enzymes [61, 98]. Our current findings showed that BA triggered increased caspase-3 activity in NP69 and TWO4 cells. This suggests that this effector caspase is playing a role in the execution of BA-induced apoptosis. Indeed, BA-induced apoptosis has been shown to be caspase-3-dependent in human colon adenocarcinoma cells [61] and rat hepatocyte [98]. In this study, caspase-3 activity was significantly reduced by Z-DEVD-FMK in NP69 and TWO4 cells treated with BA at neutral and acidic pH. Z-DEVD-FMK is a synthetic caspase-3 inhibitor which has been developed based on the substrate cleavage site of caspase-3. It acts as a pseudosubstrate for caspase-3 and is therefore a competitive inhibitor [106]. By employing a positional scanning synthetic combinatorial library, the optimal peptide recognition motif for caspase-3 has previously been determined to be DEVD (Asp-Glu-Val-Asp) [107]. Hence, DEVD inhibitor is the most potent caspase-3 inhibitor with the lowest inhibitory constant (*Ki*) against caspase-3 (*Ki* = 0.23 nM) [91].

In addition, our results showed that NP69 cells (normal nasopharyngeal epithelial cells) were more sensitive to BA treatment as compared with TWO4 cells (NPC cells). Thus, shorter exposures were used to treat NP69 cells to avoid cell detachment. These observations were similar with those obtained by Dvorak and colleagues where normal squamous oesophageal HET-1A cells appeared to be more sensitive to BA treatment as compared with human oesophageal adenocarcinoma Seg-1 cells [62]. The fact that TWO4 cells were less sensitive than NP69 cells to BA driven cytotoxicity could be due to some unknown cellular defects which contribute to apoptotic resistance or deregulation of cell death.

As discussed above, our data indicated that BA could trigger apoptosis at both neutral and acidic pH. Knowing that chromosome breakages occur in both apoptosis and chromosome rearrangements, we intended to identify the chromosome breaks mediated by BA-induced apoptosis. This study targeted the *AF9* gene located at 9p22 because 9p22 is a deletion hotspot in NPC [78]. Besides, the formation of the *MLL-AF9* fusion gene has been associated with acute myelogenous leukaemia (AML), acute lymphoblastic leukaemia (ALL), myelodysplastic syndromes (MDS) and therapy-related AML (t-AML) [89, 108]. Previous studies have reported two BCRs in the *AF9* gene, namely, BCR1 (within intron 4) and BCR2 (spans introns 7 and 8) [89, 90]. In the study of Strissel et al. (2000), two matrix association regions/scaffold attachment regions (MAR/SARs) have been isolated experimentally from the *AF9* gene. These MAR/SARs were denominated as SAR1 (found in intron 4) and SAR2 (encompasses exons 5 to 7). The *AF9* BCRs are bordered by these two MAR/SARs [89]. Using nested IPCR, we showed that the *AF9* gene cleavage frequencies in BA-treated NP69 and TWO4 cells were significantly higher than those in the untreated control. Our findings demonstrated that BA-induced apoptosis caused cleavages within the *AF9* BCR1. The *AF9* BCR1 is bordered by SAR1 and SAR2 (Fig. 9). It was found that the *AF9* BCRs share similar structural elements as the *MLL* BCR. These structural elements include MAR/SAR sequences, topo II cleavage sites and DNase I hypersensitive cleavage sites. The regions containing these similar structural elements have been found to serve as illegitimate recombination hot spots leading to the *MLL*/*AF9* translocations in leukaemia [89]. These previous findings are in good agreement with our current results that a few chromosome breaks were mapped within the *AF9* region that was previously reported being involved in t(9;11)(p22;q23) in an ALL patient. This chromosome translocation resulted in the formation of the *MLL*-*AF9* fusion gene in the ALL patient [GenBank:AM050804]. In addition, we detected a breakpoint which is identical with that identified in the ALL patient [GenBank:AM050804].

Previous reports suggested that the apoptotic nuclease CAD mediates DNA cleavages which in turn leads to chromosome translocation in leukaemia [73, 109]. Our previous findings have also suggested that CAD participates in DNA cleavages in normal nasopharyngeal epithelial cells (NP69) and NPC cells (HK1) that are undergoing oxidative stress-induced apoptosis. These cleavages may subsequently lead to chromosome rearrangement in NPC [77]. Given the involvement of oxidative stress induction in BA-induced apoptosis, it is possible that the chromosome breaks mediated by BA-induced apoptosis are also CAD-dependent. Therefore, in the current study, the possible involvement of CAD in mediating BA-induced chromosome breaks was investigated.

In non-apoptotic condition, CAD exists naturally in the cytoplasm as a heterodimer with its inhibitor, ICAD. When there is an apoptotic inducer, caspase-3 will be activated and cleaves the ICAD which possesses two caspase-3 cleavage sites. Subsequently, CAD will be released from its chaperone ICAD and enters the nucleus to cleave DNA by generating double-stranded breaks [67, 68]. An in vitro CAD assay has been conducted by Wolf and colleagues (1999) to study the roles of caspases-3, − 6, − 7, − 8, and granzyme B in mediating ICAD inactivation and apoptotic DNA fragmentation. It was found that only the DEVD-cleaving caspases, namely caspase-3 and caspase-7, inactivated ICAD and induced DNA fragmentation. Their results suggested that caspase-6, − 8 and granzyme B caused ICAD inactivation and DNA fragmentation in an indirect manner, presumably via activation of caspase-3 and/or caspase-7. They further demonstrated that caspase-3 was more efficient than caspase-7 in promoting ICAD inactivation and DNA fragmentation. Furthermore, in caspase-3 null MCF7 cells (breast cancer cell line) and cytosolic extracts, caspase-7 was found to be unable to promote ICAD inactivation in its endogenous level. Their findings concluded that caspase-3 is the main player in ICAD inactivation and apoptotic DNA fragmentation [110].

**Fig. 10:**
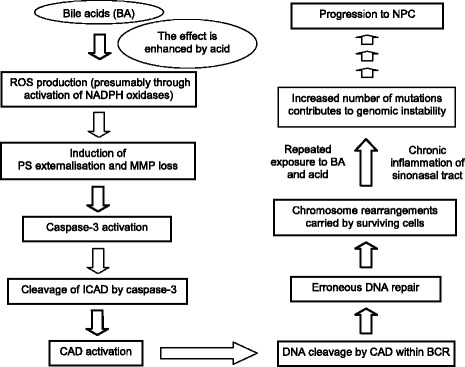
A proposed model for BA-induced apoptosis in mediating chromosome rearrangements in NPC. Exposure of nasopharyngeal epithelial cells to BA triggers intracellular ROS generation. The effect of BA in triggering ROS production is enhanced by acid through synergistic mechanisms. Increased ROS level induces PS externalisation and MMP loss. The former is acting as the apoptotic signalling which activates the downstream cascade, whereas the latter leads to MPT which in turn provokes the release of proapoptotic proteins such as cytochrome *c*. Subsequently, the main effector, caspase-3 is activated and cleaves the ICAD which possesses two caspase-3 cleavage sites. Once CAD is released from its chaperone ICAD, it enters the nucleus to cleave the DNA and causes DNA fragmentation. Cells may evade apoptosis and recover upon DNA repair. However, imprecise DNA repair may cause chromosome rearrangements in surviving cells. Repeated exposure to BA and acid (i.e. gastric duodenal refluxate) may increase the rate of mutations. The genomic instability may be exacerbated by chronic inflammation of sinonasal tissue which is repeatedly exposed to gastric duodenal refluxate. This is due to the fact that ROS production, apoptotic signalling and DNA damage may also be provoked by the inflammatory response. The cytotoxicity and genotoxicity of BA, especially in combination of acid, may therefore contribute to chromosome rearrangements in NPC

In the present study, we studied the role of CAD in mediating DNA cleavages in BA-induced apoptosis by inhibiting caspase-3 and caspase-7. As discussed above, we have clearly demonstrated that Z-DEVD-FMK could effectively inhibit the activity of caspase-3 and caspase-7. Therefore, if CAD is the main player in mediating chromosome breaks in BA-induced apoptosis, Z-DEVD-FMK treatment should reduce the number of chromosome breaks. Our data demonstrated that inhibition of caspase-3, which indirectly inhibits CAD, significantly reduced gene cleavage frequency in BA-treated NP69 cells. These findings suggest that BA induced chromosome breaks in a caspase-3-dependent manner. Given that caspase-3 is the main executioner caspase which activates CAD to cause apoptotic DNA fragmentation, thus CAD is most likely responsible for BA-induced chromosome breakages. Combining the findings from the current study and earlier work, we propose a model for BA-induced apoptosis in mediating chromosome breakages leading to chromosome rearrangements in NPC (Fig. 10). This model might also be applied to other types of cancers which have been linked to GORD or GORD-associated inflammatory diseases. These cancers include stomach, oesophageal, laryngeal, pharyngeal and lung cancers.

We note that direct association between GORD and NPC requires further investigation. Nevertheless, there is clear evidence that CRS has a positive association with an increased risk of developing NPC, and that GOR is one of the major aetiological factors of CRS. Early diagnosis of NPC is important for successful therapeutic intervention. Moreover, identification of NPC risk factors is crucial for risk prediction as well as prevention. Since the typical reflux symptoms (heartburn and regurgitation) are usually absent in patients with EOR, most of these patients are unaware of the acid refluxate exposure. Hence, patients with CRS and/or otitis media should be subjected to diagnosis for EOR such as nasopharyngeal pH monitoring. This is especially so for those who are unresponsive to conventional therapies. As a preventive measure, acid suppression therapy with antioxidant supplementation may be recommended for patients with EOR-related chronic inflammation of sinonasal tract. This may help in preventing the cytotoxicity and genotoxicity driven by both BA and acid.

## Conclusions

In summary, our study is the first to demonstrate that BA could induce apoptosis, ROS generation, MMP loss and caspase activation in nasopharyngeal epithelial cells. The effect of BA was amplified by acidic pH, suggesting that there is a synergistic effect between acid and BA in the induction of these events. We further demonstrated that BA-induced apoptosis could lead to chromosome cleavages within the *AF9* BCR. Besides, the apoptotic nuclease CAD could be a crucial player in mediating chromosome breakages in BA-induced apoptosis. Although apoptosis can act as a defence against cancer by removing cells harbouring DNA damage, cells may recover from apoptosis via DNA repair systems. However, erroneous DNA repair may cause chromosome rearrangements in surviving cells. Therefore, we speculate that repeated exposure of nasopharyngeal epithelium to BA and acid (i.e. a mixture of gastric duodenal refluxate) causes not only chronic inflammation but also elevates the risk of chromosomal alterations leading to NPC development. The increased genomic instability may be exacerbated by chronic inflammatory conditions that also induce ROS generation and DNA damage. It is also reasoned that the inflammatory conditions predispose the nasopharyngeal mucosa to transformation on repeated exposure to BA and acid. The identification of high-risk group makes early recognition and prevention of NPC a possible task. Nevertheless, further epidemiological studies of nasopharyngeal reflux and NPC are warranted.

## Additional file

Additional file 1: Flow chart depicting the simplified DNA manipulation steps in preparation for nested IPCR. (PDF 64 kb)

## Abbreviations

ALL: Acute lymphoblastic leukaemia
AML: Acute myelogenous leukaemia
BA: Bile acid
BCR: Breakpoint cluster region
CAD: Caspase-activated deoxyribonuclease
CI: Caspase inhibitor
CPT: Camptothecin
CRS: Chronic rhinosinusitis
DCFH-DA: 2′,7′-dichlorofluorescein diacetate
EBV: Epstein-Barr virus
ENT: Ear, nose and throat
EOR: Extraoesophageal reflux
FITC: Annexin V-fluorescein isothiocyanate
GORD: Gastro-oesophageal reflux disease
H_2_O_2_: Hydrogen peroxide
ICAD: Inhibitor of caspase-activated deoxyribonuclease
IPCR: Inverse polymerasechain reaction
*Ki*: Inhibitory constant
MAR/SAR: Matrix association region/scaffold attachment region
MDS: Myelodysplastic syndromes
*MLL*: Mixed lineage leukaemia
MMP: Mitochondria membrane potential
MPT: Mitochondrial permeability transition
NADPH: Nicotinamide adenine dinucleotide phosphate
NPC: Nasopharyngeal carcinoma
PI: Propidium iodide
PS: Phosphatidylserine
ROS: Reactive oxygen species
SDS: Sodium dodecyl sulfate
t-AML: Therapy-related acute myelogenous leukaemia
VP16: Etoposide
