# Supplementary material for: Bile acids at neutral and acidic pH induce apoptosis and gene cleavages in nasopharyngeal epithelial cells: implications in chromosome rearrangement
Source: BMC Cancer. 2018 Apr 12;18:409. doi: 10.1186/s12885-018-4327-4 (PMC5898073; doi:10.1186/s12885-018-4327-4)
Supplement: Supplementary file 1 — Flow chart depicting the simplified DNA manipulation steps in preparation for nested IPCR. (PDF 64 kb) [file 12885_2018_4327_MOESM1_ESM.pdf]

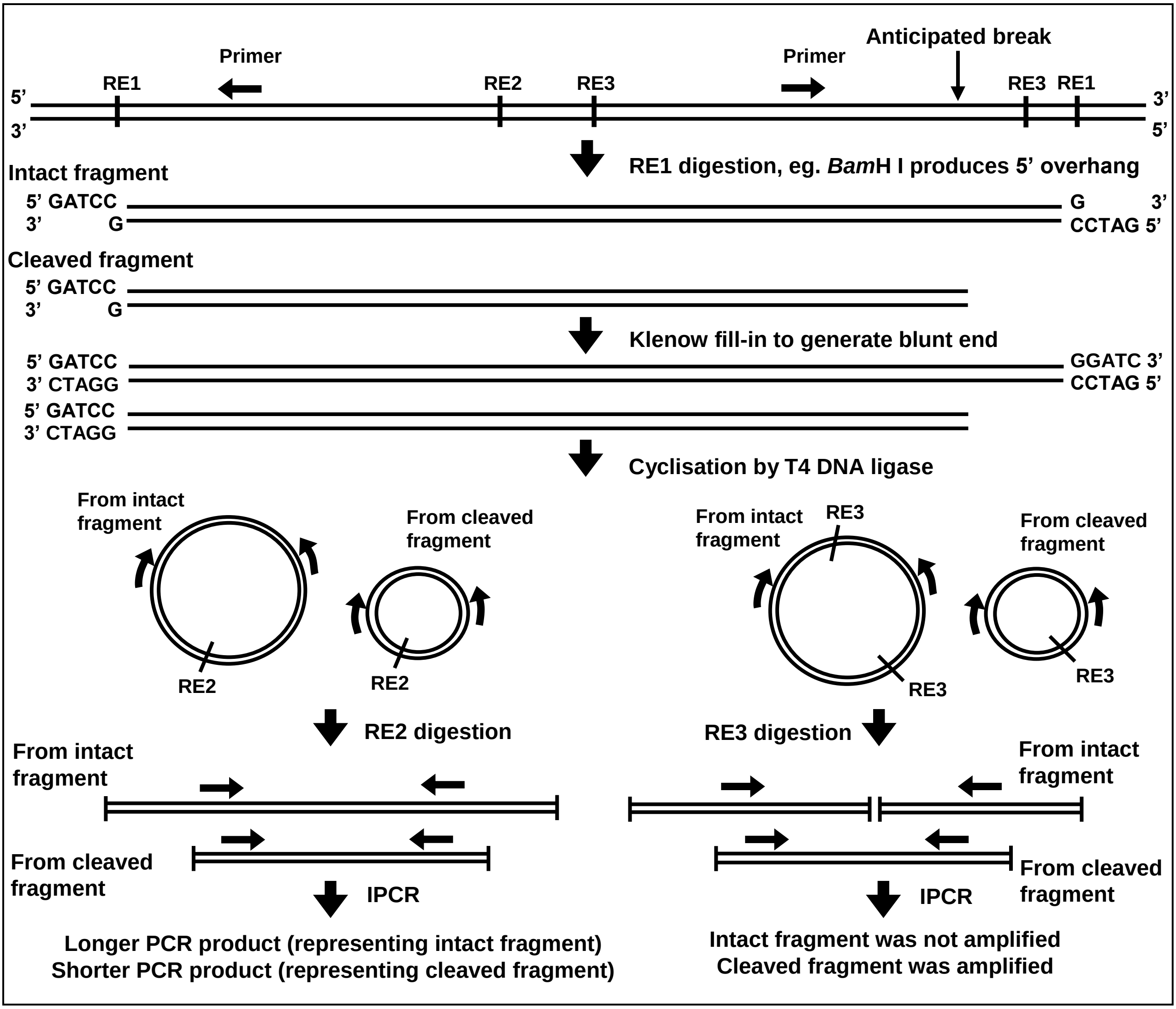

Additional file 1

Flow chart depicting the simplified DNA manipulation steps in preparation for nested IPCR. Prior to IPCR, the extracted DNA was manipulated through restriction enzymes digestion, Klenow fill-in and ligation [77].
